# Drug-Related Problems in Prescribing for Pediatric Outpatients in Vietnam

**DOI:** 10.3390/healthcare9030327

**Published:** 2021-03-14

**Authors:** Thao H. Nguyen, Vy T. T. Le, Dung N. Quach, Han G. Diep, Nguyet K. Nguyen, Anh N. Lam, Suol T. Pham, Katja Taxis, Thang Nguyen, Phuong M. Nguyen

**Affiliations:** 1 Department of Clinical Pharmacy, School of Pharmacy, University of Medicine and Pharmacy at Ho Chi Minh City, Ho Chi Minh City 700000, Vietnam; huongthao0508@gmail.com; 2 Depatment of Pharmacy, Can Tho Children’s Hospital, Can Tho City 900000, Vietnam; thanhvy121192@gmail.com; 3 Department of Pharmacology and Clinical Pharmacy, Can Tho University of Medicine and Pharmacy, Can Tho City 900000, Vietnam; dsdung1504@gmail.com (D.N.Q.); dgiahan6002@gmail.com (H.G.D.); kimnguyetnguyen@gmail.com (N.K.N.); ptsuol@ctump.edu.vn (S.T.P.); nthang@ctump.edu.vn (T.N.); 4 Department of Epidemiology, Can Tho University of Medicine and Pharmacy, Can Tho City 900000, Vietnam; lnanh@ctump.edu.vn; 5 Groningen Research Institute of Pharmacy, University of Groningen, 9713 AV Groningen, The Netherlands; k.taxis@rug.nl; 6 Department of Pediatrics, Can Tho University of Medicine and Pharmacy, Can Tho City 900000, Vietnam

**Keywords:** drug-related problems, outpatients, pediatric, prescribing, Vietnam

## Abstract

Background: Our study was conducted to determine the prevalence of drug-related problems (DRPs) in outpatient prescriptions, the impact of DRPs on treatment efficacy, safety, and cost, and the determinants of DRPs in prescribing for pediatric outpatients in Vietnam. Methods: A retrospective cross-sectional study was conducted on pediatric outpatients at a pediatric hospital in Can Tho, Vietnam. DRPs were classified according to the Pharmaceutical Care Network Europe classification (PCNE) of 2020. The study determined prevalence of DRPs and their impacts on efficacy, safety, and cost. Multivariate regression was used to identify the determinants of DRPs. Results: The study included 4339 patients (mean age 4.3, 55.8% male), with a total of 3994 DRPs, averaging 0.92 DRP/prescription. The proportion of prescriptions with at least one DRP was 65.7%. DRPs included inappropriate drug selection (35.6%), wrong time of dosing relative to meals (35.6%), inappropriate dosage form (9.3%), inappropriate indication (7.1%), and drug-drug interactions (0.3%). The consensus of experts was average when evaluating each aspect of efficiency reduction, safety reduction, and treatment cost increase, with Fleiss’ coefficients of 0.558, 0.511, and 0.541, respectively (*p* < 0.001). Regarding prescriptions, 50.1% were assessed as reducing safety. The figures for increased costs and decreased treatment effectiveness were 29.0% and 23.9%, respectively. Patients who were ≤2 years old were more likely to have DRPs than patients aged 2 to 6 years old (OR = 0.696; 95% CI = 0.599–0.809) and patients aged over 6 years old (OR = 0.801; 95% CI = 0.672–0.955). Patients who had respiratory system disease were more likely to have DRPs than patients suffering from other diseases (OR = 0.715; 95% CI = 0.607–0.843). Patients with comorbidities were less likely to have DRPs than patients with no comorbidities (OR = 1.421; 95% CI = 1.219–1.655). Patients prescribed ≥5 drugs were more likely to have DRPs than patients who took fewer drugs (OR = 3.677; 95% CI = 2.907–4.650). Conclusion: The proportion of prescriptions in at least one DRP was quite high. Further studies should evaluate clinical significance and appropriate interventions, such as providing drug information and consulting doctors about DRPs.

## 1. Introduction

Drugs play an important role in treating diseases and improving health. However, inappropriate drug use can lead to the occurrence of drug-related problems (DRPs). According to the Pharmaceutical Care Network Europe (PCNE), DRPs are situations involving drug treatment that will actually or probably affect the desired treatment outcome [1]. DRPs could be identified by comparing the appropriateness of indications, dosage form, dose, drug administration time, and drug interactions with commonly used recommendations (Table 1). DRPs can occur in various patient populations and ages. In particular, the appearance of DRPs in pediatric patients has become a major concern, with about 30 to 40% of pediatric patients experiencing at least one DRP [2]. Most DRPs are related to prescribing: drug selection, dosage, and usage [3,4]. DRP occurrence may result in treatment failure, and increase the rate of follow-up visits and rehospitalization, as well as significantly increase the need to prescribe additional drugs and the treatment costs [5]. To provide appropriate solutions to minimize DRPs and achieve desired outcomes at optimal costs, it is vital to identify and classify DRPs [6].

Children always need special health care due to their vulnerable features, thus gaining more medical attention than adults. However, because of the complexity of pediatric cases’ treatment, the DRPs rate in some developing countries was surprisingly high, reported from 31.57% to 80.1% [2,3,5]. Despite the fact that DRPs in outpatients are very common and contribute to iatrogenic morbidity [7], studies have accessed DRPs in the inpatient pediatric setting [2,8,9], and little is known about this outpatient rate [10], particularly in Vietnam. Additionally, the impacts of DRPs occurring in outpatients and inpatients are the same in terms of treatment efficacy, safety, and cost. However, since the medications are mostly administered to a child and tracked by parents and caregivers at home or school, it is difficult to determine and subsequently monitor the harmful consequences of DRPs in pediatric outpatient treatment [10,11,12]. Therefore, taking the initiative to detect and prevent these DRPs is basically needed. For this reason, this study has been conducted in which the aim is to evaluate the rate of prevalence and impact of DRPs on treatment effectiveness, safety, cost, and factors related to DRP occurrence when prescribing for pediatric outpatients.

## 2. Materials and Methods

### 2.1. Study Population and Study Design

We conducted a cross-sectional study on insured pediatric outpatients from 0 to 16 years old at a children’s hospital in Can Tho City, Vietnam. In January 2020, we retrospectively selected outpatient children’s first-time prescriptions from the hospital’s prescription database. We excluded prescriptions made on follow-up visits or those consisting only of drugs for external use during procedures or minor surgery.

### 2.2. Materials and Procedures

We used PCNE classification (version 9.1) to determine DRPs in the prescriptions [1]. DRPs were identified by comparing the appropriateness of indications, dosage form, dose, drug administration time, and drug interactions (Table 1) with commonly used recommendations in the study hospital as follows: instruction manual; Pediatric Treatment Regimen (outpatient part) [13,14]; guidelines for diagnosis and treatment of a number of diseases in children by the Ministry of Health [15] and the National Pharmacopoeia of Vietnam [16]. In addition, the assessment of DRPs was based on additional references such as the British National Formulary for Children [17]; Micromedex [18]; UptoDate [19]; Medscape [20]; and Drugs.com [21]. Any non-conformity was to be recorded as a DRP. In case of differences among recommendations, conformity with one of the recommendations was considered an appropriate prescription.

To determine the effects of DRPs on treatment effectiveness, safety, and cost, an expert team including four doctors and three pharmacists who work in and outside the hospital with at least 15 years of clinical pharmacy and pediatrics experience participated in this study. Identified DRPs were described according to each active ingredient with similar problems described and evaluated collectively. For each DRP identified, the experts independently accessed on each aspect by determining whether the identified DRP would cause (1) efficiency reduction of treatment; (2) safety reduction; and (3) treatment cost increase or not on a 4-point Likert scale with the following levels: 0—disagree, 1—partly agree, 2—agree, and 3—strongly agree. According to the interpretation of Landis and Koch (1978), levels of consensus were determined as follows: poor (κ < 0.01), weak (κ = 0.01–0.20), low (κ = 0.21–0.40), moderate (κ = 0.41–0.60), strong (κ = 0.61–0.80), and complete (κ = 0.81–1.00) [22]. The expert responses on each effect (efficiency reduction, safety reduction, and cost increase) were divided into disagreement (0 point) and agreement (1–3 points). Accordingly, a problem was concluded to have the impact when more than 50% of experts expressing agreement.

We also identified the association between DRP occurrences and determinants such as sex, age group (≤2 years; >2 years to ≤6 years; >6 years), diagnosed primary disease, comorbidity status (yes, no), and number of drugs in the prescription (<5 drugs and ≥5 drugs).

### 2.3. Statistical Analysis

We analyzed data using Microsoft Excel 2019 and SPSS 26.0. Qualitative variables were described in terms of frequency and percentage. Quantitative variables were expressed as mean ± standard deviation. We used a consensus test with Fleiss’ kappa (κ) coefficient to evaluate the level of consensus across two assessors and binary logistic regression with odds ratio (OR) with 95% confidence interval (CI) to assess the impacts of DRP determinants. We considered *p* < 0.05 to be statistically significant.

### 2.4. Ethical Consideration

The study was approved by the Medical Ethics Council of Ho Chi Minh University of Medicine and Pharmacy. Information of participating patients was kept confidential.

## 3. Results

### 3.1. Characteristics of the Study Population

The mean age of patients was 4.3 ± 3.6 years. The highest proportion of patients, 44.6%, were between >2 and ≤6 years old. Male patients accounted for 55.8%, and 78.2% of patients had respiratory diseases. The proportion of patients with no comorbidities, and patients receiving fewer than five drugs, were 80.5% and 85.3%, respectively. Characteristics of the study population are presented in Table 2.

### 3.2. DRPs in Prescribing

A total of 3994 DRPs were detected in 4339 patients, with a mean of 0.92 ± 0.8 DRP per patient. At least one DRP was identified in 2851 patients (65.7%). Dose selection and faulty time of dosing relative to meals were the most common DRPs (each accounted for 35.6%). The classification of DRPs among patients studied is shown in Table 3. Types of prescribed drugs associated with DRPs according to Anatomical Therapeutic Chemical (ATC) classification and prescribed active ingredients associated with DRPs were shown in Appendix A and Appendix B, respectively.

### 3.3. Consensus of Experts

A total of 3994 DRPs were described into 32 problems. These 32 problems were assessed by experts on the effects in efficiency, safety, and cost with a moderate degree of consensus (consensus is considered moderate when κ = 0.41–0.60). The consensus was statistically significant (*p* < 0.001), with Fleiss’ kappa coefficients for efficiency, safety, and cost at 0.558, 0.511, 0.541, respectively, as shown in Table 4.

### 3.4. Impacts of DRPs on Treatment Effectiveness, Safety, and Cost

Prescriptions with DRPs that reduced safety were found to make up the highest proportion (50.1%), followed by the proportion that increased cost (29.0%). The smallest proportion was prescriptions with DRPs that reduced treatment effectiveness (23.9%). These results were shown in Table 5. Each prescription with DRP affected one or more aspects of effectiveness, safety, and cost, shown in Appendix C.

### 3.5. Determinants of DRPs in Prescribing

Patients who were ≤2 years old were more likely to have DRPs than patients aged between 2 and 6 years old (OR = 0.696; 95% CI = 0.599–0.809) and patients aged over 6 years old (OR = 0.801; 95% CI = 0.672–0.955). Patients who had other diseases were less likely to have DRPs than patients suffering from respiratory system diseases (OR = 0.704; 95% CI = 0.604–0.820). Patients with comorbidities were less likely to have DRPs than patients with no comorbidities (OR = 0.715; 95% CI = 0.607–0.843). Patients prescribed ≥5 drugs were more likely to have DRPs than patients who took fewer drugs (OR = 3.677; 95% CI = 2.907–4.650). DRP determinants in prescribing are presented in Table 6.

## 4. Discussion

The research has become the basis of application for pharmaceutical activities in hospitals. DRPs occurrence in the study was quite high (65.7% of prescription with at least one DRP), which significantly caused safety reduction (50.1% of cases). Inappropriate drug selection and wrong time of dosing relative to meals were the most common (both accounted for 35.6%). We reported several determinants which registered that much concern needs to be placed on the care of young children (especially under 2 years of age), with respiratory disease or no-comorbidity and taking ≥5 medications. Doctors should follow treatment guidelines more strictly to avoid prescribing unnecessary drugs for Vietnamese pediatric patients.

Male patients were higher in number than female patients, with a rate of about 1.2. This rate was similar to that in most DRP studies in children [5,23,24]. Patients younger than 6 years old accounted for about 80% of participants, and their mean age was 4.3 ± 3.6. The physiological characteristics and body development in these age groups closely resembled those found between male and female patients, indicating that sex did not affect the risk of DRP occurrences in children [2]. Among the ICD-10 groups, the highest proportion had respiratory diseases. This is also common in both pediatric outpatients and inpatients in Vietnam.

Of patients with at least one DRP, 65.7% had an average of 0.92 ± 0.8 DRP per prescription; this was higher than results in previous studies, which reported 35.6% and 31.6% [3,5]. However, our proportion was lower than that of Jafarian et al., who reported that 80.1% of patients had at least one DRP [2]. Such disparities may be due to study population, differences in prescribed drugs, classifications, and reference resources used to determine DRPs. In particular, the above studies were performed on inpatients, whereas our study involved outpatients. Moreover, the criteria used to evaluate DRPs differed among studies. Finally, our higher rate of DRPs may be because our study included the time when medication was taken, which was excluded in the others.

The most common DRPs were inappropriate dose selection and wrong time of dosing relative to meals, with 35.6% for both categories. In line with previous studies, inappropriate dose selection was common in children because of their specific pharmacokinetics, age groups, and weight [4,5,24,25]. It is therefore important to calculate dose based on the age and weight of each patient. In terms of inappropriate dosage, the proportions of drug doses that were too high and those that were too low were 21.9% and 15.0%, respectively. This result differed from results of Taher et al. regarding outpatient prescriptions, which reported similar numbers of dose-related errors for high and low dose cases [26]. Dose timing relative to meals was reported as a DRP when the instructions were missed or inappropriate. We found proton pump inhibitors (PPIs), domperidone, and prednisolone to be three substances common to this DRP group. However, as previous studies did not mention this type of DRP, it was difficult to make comparisons.

The remaining groups of DRPs accounted for a relatively low proportion. Inappropriate dosage form occurred in 9.3% of patients, which was higher compared to the previous study, with 7.8% [24]. Many different dosage forms of the same active ingredient existed, to suit each age group of children. Therefore, it is necessary to choose the appropriate dosage form for each child to achieve bioavailability and avoid inappropriate dosing. The following DRP was inappropriate indication, with 7.1% (inappropriate indication for patients, and inappropriate indication for diagnosis accounted for 5.2% and 1.9%, respectively). The high proportion was because many prescribed drugs were contraindicated, or not recommended for specific patients. For example, according to the manufacturer’s instructions and pediatric treatment regimens, acetylcysteine and alimemazine are contraindicated for children younger than 2 years old. Moreover, for treating respiratory and allergic diseases, desloratadine is available for children aged 6 months, whereas loratadine should be used only for children 2 years of age and older [27]. It is recommended to use these drugs in caution with close monitoring for dosing adjustment due to risk of causing DRPs in this patient population.

Drug-drug interaction formed the lowest proportion of DRPs, with 0.3%. These cases included serious drug-drug interactions, resulting from drugs being recommended in spite of contraindications or warnings to avoid combination. Our results were similar to those reported in other studies regarding the proportion of significant drug-drug interactions recorded in outpatient prescriptions, of about 1% [24,28]. Drug-drug interaction rates were usually higher in pediatric inpatients, but with fluctuations only of around 10% [8,9,29]. The above results showed that drugs used with pediatric patients were less likely to have clinically significant drug-drug interactions.

The moderate degree of consensus among experts was determined with the narrow confidence intervals of Fleiss’ kappa coefficients when assessing the impact of DRPs on effectiveness reduction, safety reduction, and cost increase. This consensus degree was higher than that of previous studies, which had a low consensus level [30,31]. This was because we only differentiated each aspect into two levels (influence or no influence), whereas others differentiated more levels. The number of prescriptions with DRPs that reduced patient safety accounted for the highest proportion (50.1%). This result was in line with that of the study of Jafarian et al., in which DRPs related to patient safety also accounted for the highest percentage, with 43.5% [2]. This suggests that DRPs related to patient safety are common problems in treatment. Each DRP may affect more than one treatment aspect, such as therapeutic effectiveness, patient safety, and treatment cost. Specifically, DRPs related to patient safety reduction were inappropriate drug selection, drug dose too high, or wrong time of taking medication. DRPs found to decrease therapeutic effectiveness included drug dose too low, wrong time of taking medication, and drug-drug interactions. Most DRPs that increased treatment cost were related to selecting drugs that were not appropriate to diagnosis and drug dose too high.

Patients younger than 2 years were more likely to experience a higher risk of DRPs than patients between 2 and 6 years (OR = 0.696, 95% CI = 0.599–0.809) and patients older than 6 years (OR = 0.801, 95% CI = 0.672–0.955). This result differed from studies of Birarra et al. [5] and Rashed et al. [8,9] which suggested that no relationship existed between age group and risk of DRPs. This may be because DRPs resulting from contraindications or inadequate dosage form were more likely to occur in patients younger than 2 years old.

Primary diagnosis was also related to DRP occurrences. In patients with respiratory system diseases as the main diagnosis, the risk of DRP occurrences was higher than in other groups (OR = 0.704, 95% CI = 0.604–0.820). Rashed et al. expressed a conclusion that if the number of prescribed drugs per patient was ≥5 and if the patient had certain infectious and parasitic diseases, the risk of DRPs was increased [9]. In our study, we found that the majority of patients had respiratory disorders (78.2%), with four times higher than the total patient number of other diseases. Additionally, the treatment for respiratory disorders often requires the use of multiple concurrent medications in which DRPs possibly occur. For the above reasons, we paid more attention on respiratory disorders as the likelihood of DRP occurrences was higher than the other disease groups. Patients with comorbidities were less likely to develop DRPs than patients with no comorbidities (OR = 0.715, 95% CI = 2.907–4.650). This finding differed from inpatient studies, where the increase in number of diseases was associated with an increased risk of DRP occurrences [5]. However, we found that patients with comorbidities generally did not take more drugs than their counterparts, whereas for patients infected specifically with respiratory disorders (pharyngitis, allergic rhinitis, etc.), often many drugs were prescribed, such as antibiotics, H1 antihistamines, expectorants, and herbal drugs. The number of drugs used to treat respiratory diseases was thus higher than those prescribed for other patients: for those with chickenpox and calcium deficiency, only two types of drugs were prescribed, such as antiviral drugs and calcium supplements. Additionally, more than 50% of patients in our study had respiratory diseases, which by comparison led to an indication of lower risk of DRP occurrences in patients with comorbidities.

Patients receiving ≥5 drugs were at higher risk of DRPs than those receiving fewer drugs; this finding confirmed the results of most previous studies in both pediatric and adult patients [3,5,8,9,32]. In our study, patients who took ≥5 drugs were more likely to develop DRPs than patients who used fewer drugs (OR = 3.677; 95% CI = 2.907–4.650). The greater the number of drugs in a prescription, the greater the likelihood that more DRPs will occur, since each drug may cause one or more different types of DRPs. Therefore, to limit prescription of unnecessary drugs, more attention should be paid to treatment instructions. Additionally, pharmacists play an important role in the optimization of drug treatment for the patient’s benefit.

The identification of underlying factors associated with DRPs may help in preventing and resolving DRPs in pediatric patients. Therefore, the early identification of DRPs and factors associated with them may help to prevent and resolve DRPs in pediatric patients and thus enhance the most appropriate drug treatment and a more cost-effective pharmaceutical care.

Our study had several limitations. We collected only prescriptions using health insurance of outpatients. For further studies, it might be necessary to investigate physician prescriptions for fee-based patients to identify and evaluate DRPs on a more general basis. For our study, experts with experience in clinical and pediatric pharmacology identified impacts of DRPs on effectiveness, safety, and cost, but did not evaluate the levels of these impacts. The research focused on the prevalence of DRPs and their potential impacts. Future studies might evaluate clinical consequences and select appropriate evidence-based interventions for DRPs. The determination of DRPs was based on a variety of domestic and international references. The determinants of DRPs identified were: age under 2 years, main disease involving respiratory system, having no-comorbidities, and taking ≥ 5 prescribed drugs. Further research may consider surveying other factors, such as characteristics of doctors, and comorbidities.

## 5. Conclusions

Our study was the first to evaluate DRP impacts on treatment effectiveness, safety, and cost in pediatric patients in Vietnam. The percentage of pediatric prescriptions related to DRPs was quite high. Inappropriate dosing selection, wrong time of dosing relative to meals were the most common DRPs in our study. DRPs affect patient safety the most. Determinants for developing DRPs were age under 2 years, main disease involving respiratory system, having no-comorbidities, and taking ≥ 5 prescribed drugs. Future studies may identify other potential DRP determinants and lead to appropriate interventions to improve prescribing practice for pediatric patients in Vietnam. The present result underscored the need to promote pharmaceutical care at all levels of health care, especially in pediatric patients’ management to eliminate DRP and improve treatment outcomes. The involvement of clinical pharmacists in pediatric patient follow-up units is very important to reduce DRPs and they should work in collaboration with other health care professionals.

## Figures and Tables

**Table 1:** Criteria for drug-related problems (DRPs) identification.

Drug-Related Problem	Definition
Indication	
Inappropriate indication for diagnosis	Prescribed drug is not consistent with the drug indications and not intended to treat the symptoms of the diagnosed disease.
Inappropriate indication for patients	Prescribed drug is absolutely contraindicated in patients with specific age or disease. The drug is not contraindicated, but its safety and efficacy for children have not been established, and the dosage range is not recommended. However, it is still prescribed while there are other available and optimal active ingredients for the patient.
Dosage form	The dosage form is unsuitable for children since it is not possibly or accurately divided into smaller doses.
Dose selection	
Drug dose too high	The drug is prescribed with high single dose and/or excessive number of times taking medicine daily, resulting in a 24-h dose that is higher than the maximum recommended dose.
Drug dose too low	The drug is prescribed with low single dose and/or excessive number of times taking medicine daily, resulting in a 24-h dose that is lower than the minimum recommended dose.
Dose timing relative to meals	The drug is recommended to be used before meals, during meals or after meals, but the prescription is insufficiently or incorrectly instructed.
Major interaction	Severe drug-drug interactions are contraindicated or advised to avoid combination.

**Table 2:** Characteristics of the study population.

Characteristics	Number (*n* = 4339)	Percentage (%)
Age		
Mean ± standard deviation	4.3 ± 3.6	
≤2 years	1402	32.3
>2 years to ≤6 years	1934	44.6
>6 years	1003	23.1
Gender		
Male	2423	55.8
Female	1916	44.2
Main disease		
Respiratory system	3393	78.2
Other diseases	946	21.8
Comorbidities		
No	3492	80.5
Yes	847	19.5
Number of drugs in prescription		
<5 drugs	3703	85.3
≥5 drugs	636	14.7

**Table 3:** DRPs in prescribing.

DRPs	Number (*n* = 4339)	Percentage (%)
DRP proportion		
At least one DRP	2851	65.7
1 DRP	1884	43.4
2–5 DRPs	967	22.3
Mean ± standard deviation	0.92 ± 0.8	
DRP classification		
Indication	305	7.1
Inappropriate indication for diagnosis	81	1.9
Inappropriate indication for patients	224	5.2
Dosage form	403	9.3
Dose selection *	1543	35.6
Drug dose too high	950	21.9
Drug dose too low	650	15.0
Dose timing relative to meals	1546	35.6
Drug interaction	11	0.3

* Each prescription may contain one or more DRPs. The percentage of the large group may be lower than or equal to the total percentage of the constituent groups.

**Table 4:** Consensus of experts on the impact of the DRPs.

DRP Impacts	Problems (*n* = 32)	Percentage (%)	Fleiss’ Kappa Coefficients k (95% CI)	*p*
Efficiency reduction *	14	41.2	0.558 (0.555–0.560)	<0.001
Safety reduction **	15	44.1	0.511 (0.509–0.514)	<0.001
Treatment cost increase ***	17	50.0	0.541 (0.539–0.544)	<0.001

* Did this problem reduce the efficiency of treatment? ** Did this problem reduce the safety of treatment? *** Did this problem increase the cost of treatment?

**Table 5:** Impacts of DRPs on effectiveness, safety, and cost in prescribing.

DRPs Impacts * (*n* = 4339)	Number (Percentage)	DRPs				
		Indication	Dosage Form	Dose Selection	Time of Taking Medication	Major Interaction
Efficiency reduction	1038 (23.9%)		x	x	x	x
Safety reduction	2173 (50.1%)	x	x	x	x	x
Treatment cost increase	1260 (29.0%)	x	x	x		x

* A problem was concluded to have the impact when more than 50% of experts expressing agreement (1–3 points) in the consensus test.

**Table 6:** DRP determinants in prescribing.

Characteristics	Total	%	DRPs		% DRPs	OR (95% CI)	*p*	OR’ (95% CI)	*p*’
			No	Yes					
Age									
≤2 years	1402	32.3	417	985	70.3	1		1.437 (1.235–1.671)	<0.001
>2 to ≤6 years	1934	44.6	711	1223	63.2	0.696 (0.599–0.809)	<0.001	1	
>6 years	1003	23.1	360	643	64.1	0.801 (0.672–0.955)	0.013	1.151 (0.978–1.355)	0.091
Gender									
Female	1916	55.8	674	1242	64.8	1			
Male	2423	44.2	814	1609	66.4	1.091 (0.959–1.241)	0.187		
Main disease									
Respiratory system	3393	78.2	1100	2293	67.8	1			
Other diseases	946	21.8	388	558	59	0.704 (0.604–0.820)	<0.001		
Comorbidities									
No	3494	80.5	1178	2316	66.3	1			
Yes	845	19.5	310	535	63.3	0.715 (0.607–0.843)	<0.001		
Number of drugs in prescription									
<5 drugs									
≥5 drugs									
	3703	85.3	1396	2307	62.3	1			
	636	14.7	92	544	85.5	3.677 (2.907–4.650)	<0.001		

CI: confidence interval; DRPs: drug-related problems; OR: odds ratio; Using Multivariable Binary Logistic Regression: variables entered were age, gender, main disease, comorbidity status, and number of drugs in prescription. Four significant determinants are presented.

## Data Availability

Data sharing not applicable.

## Appendix A

**Table A1:** Types of prescribed drugs associated with DRPs.

ATC Code	ATC Classification	Total	DRPs	% DRPs
A	Alimentary tract and metabolism	2837	569	20.0
B	Blood and blood forming organs	3	-	-
D	Dermatologicals	100	-	-
H	Systemic hormonal preparations	1495	1217	81.4
J	Anti-infective for systemic use	2731	48	1.8
N	Nervous system	1970	-	-
P	Antiparasitic products, insecticides and repellents	2	-	-
R	Respiratory system	4073	1988	48.8
S	Sensory organs	908	-	-
V	Various	1273	-	-

## Appendix B

**Table A2:** Prescribed active ingredients associated with DRPs.

DRPs Classification	Active Ingredient	Number	DRPs	% DRPs
Inappropriate indication for diagnosis	Antihistamine (alimemazine, chlorpheniramine, loratadine)	2486	10	0.4
	Proton pump inhibition (PPI) (omeprazole, esomeprazole)	69	57	82.6
	Vitamins and minerals (calcium lactate, ergocalciferole)	622	8	1.3
	*Latobacillus acidophillus*	879	7	0.8
Inappropriate indication for patients	Acetylcysteine	486	128	26.3
	Alimemazine	691	99	14.3
	Budesonide (nasal sprays)	67	3	4.5
	Loratadine	303	6	2.0
	Racecadotril	61	2	3.3
Dosage form	Bromhexine	481	195	40.5
	Domperidone	306	174	56.9
	Montelukast	120	49	40.8
Drug dose too high	Bromhexine	481	43	8.9
	Chlorpheniramine	1492	758	50.8
	Desloratadine	34	11	32.4
	Fluticasone furoate	25	14	56.0
	Loratadine	303	133	43.9
Drug dose too low	Acetylcysteine	486	341	70.2
	Acyclovir	15	11	73.3
	Bromhexine	481	172	35.8
	Erythromycin	165	32	19.4
	Loratadine	303	27	8.9
	Racecadotril	61	48	78.7
	Salbutamol	169	48	28.4
Dose timing relative to meals	Diosmectite	176	164	93.2
	Domperidone	306	147	48.0
	PPI (esomeprazole, omeprazole)	69	22	31.9
	Metronidazole	5	5	100
	Prednisolone	1492	1217	81.6
	Simethicone	135	52	38.5
Drug interaction	Cefuroxime + PPI (esomeprazole, omeprazole)	1056	3	0.3
	Domperidone + Erythromycin	479	8	1.7
Total of DRPs			3994	

## Appendix C

**Table A3:** Groups of DRPs’ impact on effectiveness, safety, and cost in prescribing.

DRPs Impacts (n = 4339)	Number	%
Efficiency reduction only	530	12.2
Safety reduction only	650	15.0
Treatment cost increase only	36	0.8
Efficiency reduction and safety reduction	323	7.4
Efficiency reduction and treatment cost increase	24	0.6
Safety reduction and treatment cost increase	1039	23.9
Efficiency reduction, safety reduction, and treatment cost increase	161	3.7
No affection on efficiency, safety, and treatment cost	88	2.0
Total	2851	65.7
